# Cardiac troponin I as predictor for cardiac and other mortality in the German randomized lung cancer screening trial (LUSI)

**DOI:** 10.1038/s41598-024-57889-z

**Published:** 2024-03-26

**Authors:** Francisco O. Cortés-Ibáñez, Theron Johnson, Mario Mascalchi, Verena Katzke, Stefan Delorme, Rudolf Kaaks

**Affiliations:** 1 Division of Cancer Epidemiology (C020), German Cancer Research Center (DKFZ), Im Neuenheimer Feld 280, 69120 Heidelberg, Germany; 2 Translational Lung Research Center Heidelberg (TLRC-H), The German Center for Lung Research (DZL), Heidelberg, Germany; 3 Department of Clinical and Experimental, Biomedical Sciences “Mario Serio”, University of Florence, Florence, Italy; 4 Division of Epidemiology and Clinical Governance, Institute for Study, PRevention and netwoRk in Oncology (ISPRO), Florence, Italy; 5 Division of Radiology, German Cancer Research Center (DKFZ), Im Neuenheimer Feld 581, 69120 Heidelberg, Germany

**Keywords:** Troponin, Biomarkers, Lung cancer screening, Myocardial infarction, Mortality, Risk assessment, Biomarkers, Oncology, Cancer epidemiology, Heart failure

## Abstract

Cardiac Troponin I (cTnI) could be used to identify individuals at elevated risk of cardiac death in lung cancer (LC) screening settings. In a population-based, randomized LC screening trial in Germany (“LUSI” study) serum cTnI was measured by high-sensitivity assay in blood samples collected at baseline, and categorized into unquantifiable/low (< 6 ng/L), intermediate (≥ 6–15 ng/L), and elevated (≥ 16 ng/L). Cox proportional-hazard models were used to estimate risk of all-cause and cardiac mortality with cTnI levels. After exclusion criteria, 3653 participants were included for our analyses, of which 82.4% had low, 12.8% intermediate and 4.8% elevated cTnI, respectively. Over a median follow up of 11.87 years a total of 439 deaths occurred, including 67 caused by cardiac events. Within the first 5 years after cTnI measurement, intermediate or elevated cTnI levels showed approximately 1.7 (HR = 1.69 [95% CI 0.57–5.02) and 4.7-fold (HR = 4.66 [1.73–12.50]) increases in risk of cardiac death relative to individuals with unquantifiable/low cTnI, independently of age, sex, smoking and other risk factors. Within this time interval, a risk model based on age, sex, BMI, smoking history and cTnI showed a combined area under the ROC curve (AUC) of 73.6 (58.1–87.3), as compared to 70.4 (53.3–83.5) for a model without cTnI. Over the time interval of > 5–10 years after blood donation, the relative risk associations with cTnI and were weaker. cTnI showed no association with mortality from any other (non-cardiac) cause. Our findings show that cTnI may be of use for identifying individuals at elevated risk specifically of short-term cardiac mortality in the context of LC screening.

## Introduction

Randomized trials in the USA and Europe have shown that systematic screening by low-dose computed tomography (LDCT) can reduce lung cancer (LC)-related mortality^1–5^, and many countries worldwide have started or are currently introducing LC screening programs. To ensure that the expected benefit (life years gained by averting LC death) outweighs the risk of potential harms that may result from false-positive screening tests or over-diagnosis, LC screening should be targeted exclusively to individuals who are at sufficiently high risk of having a detectable lung tumor, while still having a sufficiently long residual life expectancy to expect a meaningful gain in life years in case of early LC detection. Recommended criteria for LC screening eligibility are currently based on minimal and maximal age limits, as well as on having a long-term history of intense cigarette smoking^6–8^.

Compared to the general population, long-term heavy smokers are at increased risk of having cardiovascular events, and cardiovascular disease is a leading cause of death among individuals eligible for, and taking part in lung cancer screening^1–3,5^. Thus, while systematic lung cancer screening primary aims to reduce lung cancer mortality, it is being proposed that at same time it may provide a useful setting to identify individuals who are at elevated risk of death due to cardiovascular events^9,10^. Besides offering opportunities for the prevention of premature cardiovascular death, such complementary screening tests may also help identify individuals whose potential benefit from LDCT screening is severely reduced because of elevated risk of mortality by competing causes, limited residual life expectancy and risk of screening over-diagnosis^11,12^, for whom it should be recommended that they cease further LC screening.

The cardiac troponins cTnC, cTnT and cTnI are proteins that, in complex, are centrally involved in the excitation–contraction mechanisms in cardiac myocytes^13^. The troponins cTnT and cTnI are released into the blood stream when there is damage to the cardiac tissue, and high circulating levels are used as indication for acute myocardial infarction^14^. However, the use of high-sensitivity assays have made it possible to also correlate moderate increases in these circulating troponins to risk of cardiac and other disease outcomes^13^. Other studies in healthy adults suggest that circulating serum levels of cTnI are independently associated to age, sex and systolic blood pressure^15^. In the general population, epidemiologic studies have shown that high-sensitivity measurements for cTnI can predict risk of cardiovascular disease and related deaths^16–19^, whereas blood levels of cTnT have been associated with increased risk of death caused not only by cardiovascular diseases but also by other causes^16–18^. Additional evidence suggesting that levels of cTnI improves the risk prediction of cardiovascular event in older adults^20,21^. However, there is scarce evidence from population-representative samples of long-term heavy smokers to indicate whether cTnI measurements could be useful for identifying individuals at elevated risk of cardiac death in a lung cancer screening setting.

In context of a German lung cancer screening trial, we recently reported that a high percentage of screening participants suffered from lung function impairments (chronic obstructive pulmonary disease [COPD] or preserved ratio impaired spirometry [PRISm]), and that these respiratory conditions were associated with increased risk for death of cardiovascular and all-cause mortality^22^. We here extend these analyses with findings for mortality in association to elevated blood levels of cardiac Troponin I (cTnI)—a biomarker for the risk of major cardiac events and related deaths^16–18^—to examine whether this marker may be of use for individuals at elevated short- or medium-term risk of death by cardiac, and other causes.

## Materials and methods

### Study design and participants

The German Lung Cancer Screening Intervention Trial—LUSI^5,23^ is a registered randomized trial (ISRCTN30604390, date of registration: 19/07/2007) with a recruitment phase between October 2007 and April 2011 and active screening between October 2007 and April 2016, and with continuing prospective ascertainment of lung cancer incidence and overall mortality until to date. For recruitment of trial participants, a total of 292,000 men and women aged 50–69 years were extracted from population registers in the city of Heidelberg and surrounding communes, and were asked through mailed questionnaires about their past and current smoking habits. To be eligible for the study, participants had to accumulate ≥ 25 years of smoking an average of ≥ 15 cigarettes per day, or alternatively ≥ 30 years smoking an average of ≥ 10 cigarettes per day, while not having quit smoking more than 10 years prior to the screening invitation (similar to the criteria used for the Dutch-Belgian NELSON study^2^). Among 89,722 respondents who replied to the pre-baseline smoking questionnaire, 4708 were eligible by these criteria and willing to participate in the study, and were invited to the German Cancer Research Center (DKFZ) in Heidelberg for CT screening. A total of 4052 participants finally took part in the trial and were randomized into a screening intervention arm (n = 2029) comprising five annual CT screenings, and a control arm (n = 2023) without screening; 2007 participants in the CT arm were also offered a spirometry test to be performed on occasion of their baseline CT scan. Practically all participants are of Caucasian ethnic ancestry. A more detailed report of the study design and randomization strategy is described elsewhere^24^. For our present analyses, we excluded participants who had missing measurements for cTnI, those who had unknown vital status, abnormal FEV1/FVC ratio (> 1)^25^, or missing values for BMI (N = 399).

### Questionnaire information, at baseline and during prospective follow-up

At their first screening visit, participants completed a baseline questionnaire, then also responded to follow-up questionnaires on occasion of their annual follow-up screenings, about (recent changes in) smoking habits, use of radiologic (X-ray, CT, MRI) or other (e.g., endoscopic) examinations of the lungs independently of annual CT screening, surgical chest procedures, and possible occurrence of cancer (lung or other organs). In addition, they reported about respiratory symptoms, pulmonary symptoms, and physician-based diagnoses of cardiovascular disease (myocardial infarction, coronary heart disease, angina pectoris, and stroke), diabetes mellitus, arterial hypertension and other comorbidities.

After completion of the five screening rounds, annual follow-up questionnaires continued to be sent to participants in both study arms until to date. Participants who did not respond were contacted by telephone and were offered assistance with the completion of questionnaires during the call.

### Spirometry

In the screening arm only, 2007 (out of 2029) participants performed pre-bronchodilator spirometry using a MasterScreen IOS (VIASYS Healthcare) spirometer. For these participants, FEV1/FVC ratios were calculated from the largest FEV1 and FVC values recorded in any 1 of 2 repeated assessments. Individuals’ predicted FEV1 and FVC values for a given age, sex, body height, and race (FEV1% predicted, FVC% predicted) were calculated by previously established Eqs. ^25^. Participants with FEV1/FVC < 0.70 were classified as having COPD, and the severity of their airflow impairment was further graded into stages 1 (FEV1 ≥ 80% predicted), 2 (50% ≤ FEV1 < 80% predicted), or 3–4 (FEV1 < 50% predicted) following the GOLD criteria^26^. Participants with FEV1/FVC ≥ 0.70 but with FEV1% < 80% were classified as having PRISm.

### Laboratory methods

Plasma samples were retrieved from – 80 °C long-term storage with no freeze/thaw cycles from the time of sample collection. Samples were thawed at 4 °C and cTnI quantified at the University Hospital Heidelberg Central Laboratory, using the Siemens high sensitivity *Troponin I* (TNIH) assay on the ADVIA Centaur platform (Siemens Healthcare, Erlangen, Germany). According to manufacturer’s indications, the hs-cTnI assay’s combined 99th percentile upper reference level (URL) is 47.34 ng/L with a CV of < 5%. Gender specific 99% URLs are 57.27ng/L and 36.99ng/L for male and female subjects respectively. The Heidelberg laboratory used a 99th percentile URL of 40 ng/L in view of years’ long experience with the assays for local clinical purposes. The assay’s limit of blank (LOB) is 0.5ng/L, the limit of detection (LOD) is 1.6ng/L and the limit of quantification (LOQ) at 10%CV is 6.0ng/L while at 20%CV the LOQ is 3.0 ng/L. The ADVIA Centaur instrument was set for a precision of only 2 decimal places for these hs-cTnI measurements and raw data reported in ng/ml, where values of 0.006 ng/ml (6 ng/L) were rounded to 0.01 ng/ml, and values below 0.006ng/ml were labeled as below the quantification limit. For the remainder of this manuscript, however, we present our results from the cTnI measurements in ng/L, for ease of comparison with other studies in which high-sensitivity assays were used, and as the internationally accepted standard unit for hs-Troponin measurements. The measurement values were strongly skewed towards lower values and clustered due to rounding in original ng/ml units as follows: unquantifiable < 0.006 ng/ml (< 6 ng/L), N = 3012; 0.01 ng/ml (≥ 6–15 ng/L), N = 466; 0.02 ng/ml (≥ 16–25 ng/L), N = 71; 0.03 ng/ml (≥ 26–35 ng/L), N = 32; 0.04 ng/ml (≥ 36–45 ng/L), N = 14; 0.05 ng/ml (≥ 46–55 ng/L), N = 11; 0.06 ng/ml (≥ 56–65 ng/L), N = 2. The few remaining values (N = 45) were spread over a wide range of very high (“outlier”) values all ≥ 66 ng/L, and likely had resulted from laboratory measurement error (for this study, no duplicate measurements were made to correct for artificial outliers). In this latter series no cardiac deaths were observed.

### Prospective follow-up for mortality end-points

In the full study cohort, ninety-nine cases of lung cancer were diagnosed until April 2021 among the participants in the CT arm, of which 63 were screen-detected, 6 were “interval” cases missed by CT screening, and 30 were diagnosed after the screening period (i.e., 12 or more months after a participant’s last screening participation). In the control arm, a total of 86 LC cases were reported. Non-screen detected LC cases were identified through annual follow-up questionnaires, reports from treating hospitals, and systematic linkages to cancer and mortality registries. For all LC patients, detailed information (pathology reports, medical letters on diagnosis and treatment, radiology reports) was obtained and coded to ICD-O-3 for tumor histology and stage.

Mortality and causes of death were ascertained through record linkages with/to municipal registries (for vital status) and regional health offices (for detailed death certificates). Based on the death certificate data and clinical data records, all cases of death were classified by leading cause (ICD-10).

### Statistical analyses

Sociodemographic and health-related characteristics from the baseline in the LUSI CT trial were presented as means (SD), medians (25th/75th percentiles) or proportions, and stratified by cTnI categories. For analyses of cTnI we defined three categories: low/unquantifiable (participants with values below the quantification limit of < 6.0 ng/L, N = 3 012); intermediate (individuals with value of ≥ 6–15 ng/L, N = 466); and elevated (participants who had cTnI values ≥ 16 ng/L, N = 175).

Multivariable Cox proportional hazard models adjusted primarily by age, sex, BMI and potential key confounders: lifetime smoking duration, time since smoking cessation, and average cigarettes per day, were applied to prospectively quantify the associations of cTnI concentrations with risk of cardiac death or death by other causes. In these models, cTnI was examined either as a categorical variable, as described above, or as a continuous, quantitatively scored variable (i.e., measurements on the rounded ng/ml scale) transformed on the log_2_ scale. Log-likelihood ratio tests were used to test for the improvement in model fit with the addition of the cTnI variable to a base model for age, sex, BMI, and smoking history (lifetime smoking duration, average number of cigarettes smoked per day, time since smoking cessation for ex-smokers). Improvements in discrimination per marker added were evaluated by integrated discrimination improvement (IDI) indices, net reclassification index (NRI) and by the increase in the overall area under the receiver operator curve (AUC). Models were internally cross-validated on 1,000 bootstrapped samples to correct for overfitting.

Statistical analyses were performed using R-4.3.2, and SAS (version 9.4, SAS Institute, NC, USA).

### Informed consent and ethical approvals

All the included participants provided written informed consent for the present study. The trial was conducted in accordance with the principles of the Declaration of Helsinki and was approved by the University of Heidelberg Medical Ethics Committee (No. 073/2001) and by the radiation protection authority (BfS, 22462/2, 2006-045).

We followed and used the recommendations of the CONSORT reporting^27^.

## Results

### Participant characteristics

We considered 3653 participants who met the inclusion criteria for the present analyses; of those, 1949 were in the CT screening arm and 1704 in the control arm. A graphical representation of the study’s enrollment and screening process is in Supplemental Fig. S1. The percentage of women included was (35.2%), and the median age within the cohort was 56.2 (IQR: 49.2–70.9) (Table 1). More than 30% of the participants were ex-smokers who had stopped smoking for at least one year or more, whereas all others were current smokers. In the two study arms combined, 3012 of the 3653 study participants (82.4%) had cTnI values below the quantification limit of < 6ng/L, 466 (12.8%) had intermediate levels (≥ 6–15 ng/L), and 175 (4.8%) who had elevated cTnI measurements (≥ 16 ng/L).

**Table 1:** Characteristics of the study population in the lung cancer-screening cohort (LUSI) [Control arm + CT screening arm, by cardiac Troponin concentration categories].

	C1 (N = 3012) (< 6 ng/L)	C2 (N = 466) (≥ 6–15 ng/L)	C3 (N = 175) (≥ 16 ng/L)	*p*	Overall (N = 3653)
Sex					
Males	1862 (61.8%)	372 (79.8%)	132 (75.4%)		2366 (64.8%)
Females	1150 (38.2%)	94 (20.2%)	43 (24.6%)	< 0.001	1287 (35.2%)
Age					
Median [Min, Max]	55.7 [49.2, 70.9]	59.2 [50.1, 70.9]	57.8 [50.1, 70.2]	< 0.001	56.2 [49.2, 70.9]
Body mass index					
Median [Min, Max]	26.1 [17.0, 52.1]	26.8 [17.2, 44.3]	26.5 [18.3, 36.1]	0.001	26.2 [17.0, 52.1]
Smoking status					
Current smoker	1869 (62.1%)	279 (59.9%)	100 (57.1%)		2248 (61.5%)
Former smoker	1143 (37.9%)	187 (40.1%)	75 (42.9%)	0.314	1405 (38.5%)
Smoking duration (years)					
26–30	542 (18.0%)	73 (15.7%)	30 (17.1%)		645 (17.7%)
31–35	1005 (33.4%)	97 (20.8%)	47 (26.9%)		1149 (31.5%)
36–40	901 (29.9%)	144 (30.9%)	51 (29.1%)		1096 (30.0%)
> 40	564 (18.7%)	152 (32.6%)	47 (26.9%)	< 0.001	763 (20.9%)
Lung function categories (only in the CT screening arm)					
Normal	1070 (67.3%)	153 (60.2%)	70 (66.7%)		1293 (66.3%)
PRISm	240 (15.1%)	44 (17.3%)	17 (16.2%)	0.608	301 (15.4%)
COPD GOLD1	76 (4.8%)	15 (5.9%)	0 (0%)	< 0.001	91 (4.7%)
COPD GOLD2_4	204 (12.8%)	42 (16.5%)	18 (17.1%)	0.150	264 (13.5%)
Coronary heart disease (baseline)					
No	2781 (92.3%)	408 (87.6%)	141 (80.6%)		3330 (91.2%)
Yes	146 (4.8%)	43 (9.2%)	28 (16.0%)	< 0.001	217 (5.9%)
I don't know	73 (2.4%)	11 (2.4%)	5 (2.9%)		89 (2.4%)
Not answered	12 (0.4%)	4 (0.9%)	1 (0.6%)		17 (0.5%)
Arterial hypertension (baseline)					
No	2016 (66.9%)	234 (50.2%)	81 (46.3%)		2331 (63.8%)
Yes	898 (29.8%)	217 (46.6%)	89 (50.9%)	< 0.001	1204 (33.0%)
I don't know	91 (3.0%)	15 (3.2%)	5 (2.9%)		111 (3.0%)
Not answered	7 (0.2%)	0 (0%)	0 (0%)		7 (0.2%)
Diabetes (baseline)					
No	2723 (90.4%)	391 (83.9%)	149 (85.1%)		3263 (89.3%)
Yes	231 (7.7%)	72 (15.5%)	24 (13.7%)	< 0.001	327 (9.0%)
I don't know	44 (1.5%)	3 (0.6%)	1 (0.6%)		48 (1.3%)
Not answered	14 (0.5%)	0 (0%)	1 (0.6%)		15 (0.4%)
Myocardial infarction (self-reported)					
No	2763 (91.7%)	387 (83.0%)	141 (80.6%)		3291 (90.1%)
Previous MI event	133 (4.4%)	48 (10.3%)	19 (10.9%)	< 0.001	200 (5.5%)
Incident MI event	116 (3.9%)	31 (6.7%)	15 (8.6%)	< 0.001	162 (4.4%)
Stroke (self-reported)					
No	2878 (95.6%)	443 (95.1%)	157 (89.7%)		3478 (95.2%)
Previous stroke event	71 (2.4%)	11 (2.4%)	11 (6.3%)	0.004	93 (2.6%)
Incident stroke event	63 (2.1%)	12 (2.6%)	7 (4.0%)	0.184	82 (2.2%)

At entry into the LUSI trial, slightly over 30% of the participants reported having arterial hypertension as diagnosed by their treating physician, 2.6% and 5.5% reported (respectively) a previous stroke or myocardial infarction event, whereas 9% reported diabetes and 6% prevalent coronary heart disease. Overall, 362 reported a history of myocardial infarction, of which 200 occurred before study recruitment, and 162 during the course of the study (Table 1). Higher levels of cTnI were associated with higher percentages of participants reporting a past history of coronary heart disease (p < 0.001), myocardial infarction (p < 0.001), stroke (p = 0.001), diabetes (p < 0.001), arterial hypertension (p < 0.001) and history of long-term smoking (i.e. more than 40 years) (p =  < 0.001). In addition, higher levels of cTnI were associated with a higher percentage of participants reporting a myocardial infarction during prospective follow-up. Spirometry was performed only in the CT screening arm, where 301 participants (15.4%) showed lung function tests indicating PRISm, and 264 (13.5%) had tests indicating moderate to severe stages (GOLD stage 2–4) of COPD. The prevalence of COPD or PRISM did not vary significantly across the three levels of cTnI (Table 1).

**Figure 1:**
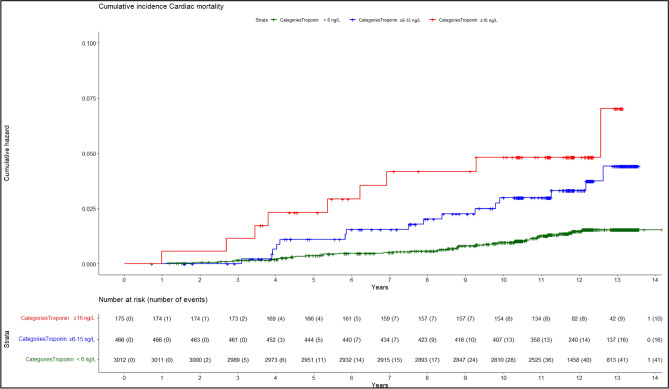
Cumulative incidence of cardiac mortality events from participants according to measurement categories of cTnI.

The median follow-up time for all 3653 participants was 11.87 years, and in that period a total of 439 of the 3653 participants died, 67 due to cardiac events. Furthermore, a total of 154 LC cases were diagnosed (92 in the CT screening arm and 70 in the control arm), and a total 84 participants died of LC (38 in the CT screening arm and 46 in the control arm). For 60 of the 67 participants who died of a cardiac event, that episode represented their first-ever cardiac attack (no other previous event registered or self-reported), whereas the remaining 7 had a self-reported history of previous myocardial infarction. For participants in the unquantifiable, intermediate and elevated categories of cTnI a Kaplan–Meier analysis (Supplemental Fig. S2) and corresponding cumulative incidence curves (Fig. 1) showed cumulative incidences for cardiac death of, respectively, 0.4% (11/3012), 1.1% (5/466) and 2.3% (4/175) within the first 5 years after blood cTnI measurement, and 0.9% (28/3012), 2.8% (13/466) and 4.6% (8/175) over the first 10 years.

### Prospective associations of cTnI with mortality risk

Focusing on the first 5 years of follow-up, proportional hazards models (adjusting for age, BMI, smoking history, arterial hypertension, history of diabetes and past (pre-baseline) occurrences of myocardial infarction or stroke), showed an approximate 4.7-fold increase (HR = 4.66 [95% CI 1.73–12.50]) in risk of cardiac death for participants with serum levels of cTnI ≥ 20 ng/L, and a close to 1.7-fold increase in risk (HR = 1.69 [CI 0.57–5.02) for participants with intermediately elevated cTnI (cTnI = 10 ng/L), relative to those with cTnI levels below the quantification limit (Table 2). The adjusted models showed weaker associations of elevated cTnI showed with risk of with cardiac deaths occurring between > 5 and ≤ 10 years after the time of blood donation (measurement of cTnI). Adjustments for spirometry-based categories of pulmonary function (COPD, PRISm) did not materially alter relative risk estimates (analyses in the CT arm only; Supplemental Table S1). Irrespective of the duration of follow-up time, models showed no association of cTnI with risk of death by non-cardiac causes (Table 2) and showed no association of cTnI with risk of either developing, or dying from LC (data not shown).

**Table 2:** Hazard ratios for all-cause mortality and cardiac mortality by categories of Troponin I (cTnI), in the German lung cancer screening trial (LUSI)^1^.

Troponin	n_cases/controls_	Complete cohort	
		HR (95% CI)	*p*_*trend*_
Follow-up ≤ 5 years			
Cardiac mortality^3^			
Category 1 (< 6.0 ng/L) ref^¶^	n = 13/2999		
Category 2 (≥ 6–15 ng/L)	n = 5/461	1.69 (0.57–5.02)	
Category 3 (≥ 16 ng/L)	n = 5/170	4.66 (1.73–12.50)	**0.005**
Categories 2 and 3 combined	n = 10/631	2.44 (1.06–5.61)	**0.035**
Troponin I ng/L (continuous)^2^	n = 23/3630	1.25 (1.05–1.49)	**0.011**
Mortality by all other causes (excluding cardiac mortality)			
Category 1 (< 6.0 ng/L) ref^¶^	n = 59/2953		
Category 2 (≥ 6–15 ng/L)	n = 17/449	1.25 (0.72–2.16)	
Category 3 (≥ 16 ng/L)	n = 7/168	1.56 (0.69–3.53)	0.209
Categories 2 and 3 combined	n = 24/617	1.64 (0.85–3.18)	0.253
Troponin I ng/L (continuous)^2^	n = 83/3570	1.09 (0.97–1.23)	0.134
Follow-up > 5–10 years			
Cardiac mortality^3^			
Category 1 (< 6.0 ng/L) ref^¶^	n = 17/2995		
Category 2 (≥ 6–15 ng/L)	n = 8/458	1.98 (0.73–5.33)	
Category 3 (≥ 16 ng/L)	n = 3/172	1.66 (0.46– 5.97)	0.998
Category 2 and 3 combined	n = 11/630	1.88 (0.78–4.53)	0.156
Troponin I ng/L (continuous)^2^	n = 28/3625	1.10 (0.92–1.31)	0.264
Mortality by all other causes (excluding cardiac mortality)			
Category 1 (< 6.0 ng/L) ref^¶^	n = 142/2870		
Category 2 (≥ 6–15 ng/L)	n = 33/433	1.09 (0.74–1.60)	
Category 3 (≥ 16 ng/L)	n = 9/166	0.88 (0.45–1.74)	0.185
Categories 2 and 3 combined	n = 42/599	1.04 (0.73–1.47)	0.821
Troponin I ng/L (continuous)^2^	n = 184/3469	1.00 (0.91–1.09)	0.976
Full follow up time, up to max. 14.1 years			
Cardiac mortality^3^			
Category 1 (< 6.0 ng/L) ref^¶^	n = 41/2971		
Category 2 (≥ 6–15 ng/L)	n = 16/450	1.58 (0.84–2.96)	
Category 3 (≥ 16 ng/L)	n = 10/165	2.71 (1.34–5.45)	**0.004**
Categories 2 and 3 combined	n = 26/615	1.88 (1.11–3.17)	**0.017**
Troponin I ng/L (continuous)^2^	n = 67/3586	1.15 (1.03–1.28)	**0.014**
Mortality by all other causes (excluding cardiac mortality)			
Category 1 (< 6.0 ng/L) ref^¶^	n = 278/2734		
Category 2 (≥ 6–15 ng/L)	n = 71/395	1.15 (0.87–1.50)	
Category 3 (≥ 16 ng/L)	n = 21/154	1.08 (0.70–1.71)	0.053
Categories 2 and 3 combined	n = 92/549	1.13 (0.89–1.44)	0.304
Troponin I ng/L (continuous)^2^	n = 370/3283	1.03 (0.97–1.09)	0.317

Significant values are in bold.

^1^Based on competing risk models adjusted by age, sex, BMI, smoking history, stroke event, myocardial infarction event (previous or incident), hypertension (self-reported), coronary heart disease and diabetes.

^2^Continuous values were log_2_ transformed.

^3^The following ICD 10 codes were included in cardiac mortality: I07.1, I11.0, I13.2, I21.0, I21.4, I21.9, I22.9, I25.1, I25.10, I25.5, I25.9, I42.0, I42.9, I46.1, I46.9, I48.9, I49.0, I49.9, I50.19, I50.9, I51.9

^¶^Categories of cTnI, as defined by the following concentrations: < 6.0 ng/L, ≥ 6–15 ng/L, ≥ 16 ng/L.

Table 3 shows the improvement in risk discrimination for cardiac mortality, for a model based on age, sex, BMI and smoking history (lifetime duration, cigarettes/day, time since quitting,) plus cTnI versus a model not including cTnI. Measured by the AUC and IDI and NRI indices, there were significant improvements in prediction of cardiac death over the first 5, as well as the first 10, years after cTnI measurement. Over the first 5 years of follow-up, the AUC for the combined model including cTnI was 73.6% (58.1–87.3) vs. 70.5% (51.6%-86.4%) for a model without cTnI; over the first 10 years of follow-up the AUC values were lower (AUC = 70.0 (60.9–81.3) vs. AUC = 68.9 (57.6–79.7), respectively ).

**Table 3:** Evaluation of discrimination performance when adding troponin I to predict risk of cardiac mortality in the German randomized lung cancer-screening trial (LUSI).

	Log-likelihood ratio test		
	(χ^2^; df; p)	IDI-NRI (95% CI)*	AUC (%) 95% CI**
Cardiac mortality			
First 5 years of follow up (n_cases_ = 23)			
(a) Model: age, sex, BMI and smoking history			70.5 (51.6–86.4)
(b) Model a + cTnI (vs. Model a)	7.80; 1; 0.010	IDI: 0.005 (0.000, 0.024)^¶^ NRI: 0.278 (0.070, 0.486)^¶^	73.6 (58.1–87.3)
First 10 years of follow up (n_cases_ = 51)			
(a) Model: age, sex, BMI and smoking history			68.9 (57.6–79.7)
(b) Model a + cTnI (vs. Model a)	11.4; 1; 0.003	IDI: 0.013 (0.002, 0.048)^¶^ NRI: 0.261 (0.100, 0.407)^¶^	70.0 (60.9–81.3)
Entire follow up time (n_cases_ = 67)			
(a) Model: Age, sex, BMI and smoking history			65.5 (53.8–74.5)
(b) Model a + cTnI (vs. Model a)	12.56; 1; 0.001	IDI: 0.014 (0.003–0.049)^¶^ NRI: 0.226 (0.081–0.372)^¶^	66.6 (56.4–75.8)

*Troponin I is included as a categorical variable (C1, C2, C3 categories).

**Models were internally cross-validated on 1000 bootstrapped samples to correct for overfitting.

*IDI* integrated discrimination index, *NRI* net reclassification index, *AUC* area under the receiver operator curve.

^¶^*p* < 0.05.

## Discussion

In this analysis of long-term heavy smokers, all participants in a LC screening trial, we found that baseline measurements of cTnI, as obtained from a high-sensitive assay by a routine clinical chemistry laboratory, can help identify individuals who are at strongly increased short- and medium-term risk for cardiac death. Using cTnI to classify screening participants into three risk categories, we found that relative to those (82%) at lowest risk, about 13% of the screening participants were at approximately 1.7-fold, and additional 5% of participants at approximately 4.7-fold higher risk of cardiac death over the next five years, independently of age, lifetime smoking history, history of past cardiac morbidity and other potential confounding factors. Over the first 10 years, the cumulative incidences for cardiac deaths in these three risk categories amounted to 0.9%, 2.8% and 4.6%, respectively. Furthermore, cTnI appeared to be a marker very specific for cardiac deaths, but not for risk of death by other causes. Importantly, the majority of subjects who died of cardiac disease had had no previous cardiovascular events, suggesting that these participants may not have been aware of being at particularly increased risk. Our findings thus show that cTnI measurements from a routine clinical biochemistry laboratory can be used to identify a smaller proportion of lung cancer screening participants at elevated risk of cardiac death (e.g., about 5 percent of participants with 10-year risk of close to 5%) who were previously unaware of being at elevated risk, and who could be offered preventive treatment.

Current research focuses on the complementary use of CT-images for identifying individuals at elevated risk of cardiovascular mortality, on the basis of radiologic ascertainments of emphysema, COPD or coronary artery calcification scores^28,29^. A limitation of our study is that for the present analyses we did not dispose of CT-based assessment of emphysema and coronary artery calcification; thus, we could not examine the added value of cTnI measurements for this purpose, compared to radiologic data. Besides arterial calcification scores, however, excess cardiac mortality has also been found to be increased among individuals with impaired spirometry—e.g. COPD^30^, or preserved ratio impaired spirometry (PRISm)^31^—a comorbidity that affects large proportions of lung cancer screening participants^9,10,22^. For the CT arm of the LUSI trial, we previously found that about a third of study participants had COPD or PRISM, and that these participants were at two- to threefold increased risks of overall, lung cancer-related and cardiac mortality relative to participants with normal lung function^22^. In our present analyses, we found that cTnI was associated with cardiac death independently of PRISm or COPD, and showed no clear relationship with either form of respiratory impairment. It thus appears that cTnI may signal higher cardiac-related mortality risk independently of impaired lung function, and similar conclusions were also drawn from analyses of cohorts of COPD patients, in which cTnI^32–34^ or cTnT^35^ were related to increased risk of cardiovascular and all-cause mortality independently of the degree of respiratory impairment.

As of 2017 there were significant improvements in the sensitivity of cTnI assays since manufacturers progressed from 4 to 5th generation assays with the LOD falling to as low as 1.3 ng/L depending on the platform and assay^36^. A general complexity of our and other cohort studies is that different analytic platforms and assay types for troponin are being used which have been developed using differing capture and detection antibody pairs and can lead to varied sensitivity and heterogeneity in the quantitative calibration of cTnI measurements^13^. This complicates the definition of standardized quantitative cut-points of cTnI assays for categorization of short- to medium-term cardiac risk.

A limitation of our study is that, in spite of using a high-sensitivity assay, the instrument used for performing the cTnI measurements had been set for a precision of only 2 decimal places on a ng/ml scale, instead of using internationally recommended ng/L units. This led to some artificial loss in precision in the cTnI measurements. A further limitation of our study is that the total number of cardiac death events in this cohort was too small to allow greater-depth analyses of risk discrimination by considering combinations of cardiovascular risk factors. Finally, in this cohort of screening participants there was no information about further cardiovascular risk variables, such as blood pressure in mm, plasma values for creatinine, or blood lipid profile, that is required for standard risk scores for the prediction of coronary events (e.g. the SCORE2) or sudden cardiac death (e.g. as in^37,38^).

In spite of the above limitations, our present results suggest that measurements of cTnI—a relatively inexpensive routine marker—may complement efforts for the identification of individuals at higher risk of a fatal cardiac event. In sum, and in combination with systematic offers to assist with smoking cessation, these efforts may help implement timely preventive treatments, so as to further reduce premature mortality among long-term smokers.

### Supplementary Information

Supplementary Information.

## Data Availability

The data used for the present study are not publicly available, but are available from the corresponding author upon reasonable request.
