# Supplementary material for: Cardiac troponin I as predictor for cardiac and other mortality in the German randomized lung cancer screening trial (LUSI)
Source: Sci Rep. 2024 Mar 26;14:7197. doi: 10.1038/s41598-024-57889-z (PMC10965973; doi:10.1038/s41598-024-57889-z)
Supplement: Supplementary file 1 — Supplementary Information. [file 41598_2024_57889_MOESM1_ESM.docx]

**Supplemental Figure 1. Inclusion and exclusion criteria from the German randomized lung cancer screening trial (LUSI).**


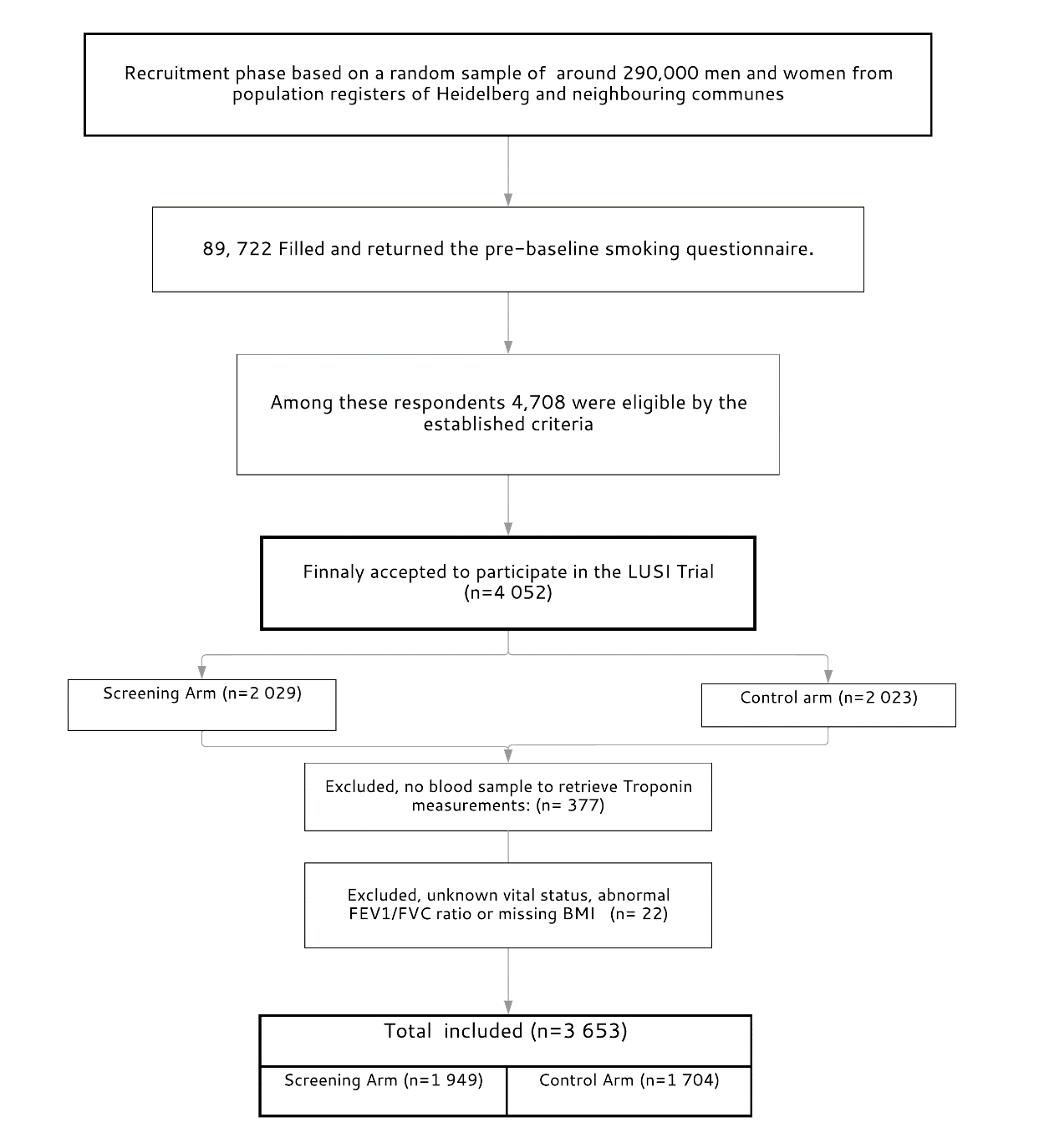


**Supplemental Figure 2. Kaplan-Meier analysis of cardiac mortality events from participants according to measurement categories of cTnI.**


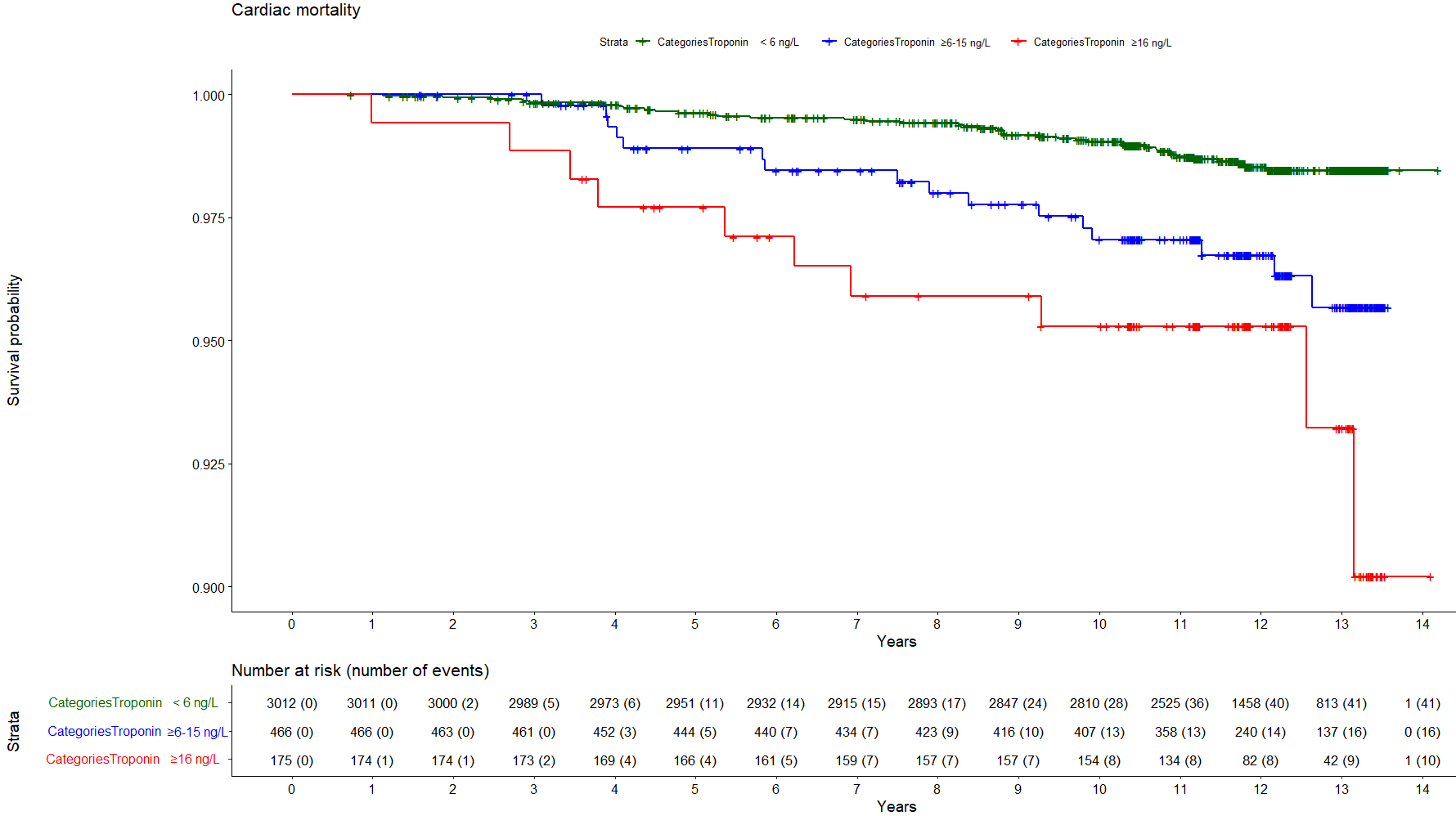


**Supplemental Table 1. Hazard ratios from competing risk analysis for all-cause mortality and cardiac mortality by categories of Troponin I (cTnI), in the CT screening arm of the German Lung Cancer screening trial (LUSI).**

|  | **n_cases/controls_** | **CT screening arm^1^** | |  |
| --- | --- | --- | --- | --- |
|  |  |  |  |  |
| **Troponin** |  | **HR (95%CI) *p_trend_*** | |  |
| **Cardiac mortality ^2^** |  |  |  |  |
| Category 1 (<6.0 ng/L) ref^¶^ | n=20 / 1570 |  |  |  |
| Category 2 (≥6-15 ng/L) | n=7 / 247 | 1.47 (0.60 – 3.57) |  |  |
| Category 3 (≥ 16 ng/L) | n=8 / 97 | 4.18 (1.91 – 9.13) | **0.001** |  |
|  |  |  |  |  |
| Category 2 & 3 combined | n=15 / 344 | 2.27 (1.15 – 4.46) | **0.031** |  |
|  |  |  |  |  |
| Troponin I ng/L (continuous) | n=35/1914 | 1.20 (1.03-1.41) | **0.017** |  |
|  |  |  |  |  |
| **Mortality by all other causes (excluding cardiac mortality)** |  |  |  |  |
| Category 1 (<6.0 ng/L) ref^¶^ | n=150 / 1440 |  |  |  |
| Category 2 (≥6-15 ng/L) | n=41 / 213 | 1.18 (0.82 – 1.71) |  |  |
| Category 3 (≥ 16 ng/L) | n=7 / 98 | 0.63 (0.30 – 1.34) | 0.746 |  |
|  |  |  |  |  |
| Category 2 & 3 combined | n= 48/ 311 | 1.05 (0.74 – 1.47) | 0.776 |  |
|  |  |  |  |  |
| Troponin I ng/L (continuous) | n=198/1751 | 0.99 (0.91-1.07) | 0.848 |  |
| **≤ 5 years** |  |  |  |  |
| **Cardiac mortality ^3^** |  |  |  |  |
| Category 1 (<6.0 ng/L) ref^¶^ | n=8 / 1582 |  |  |  |
| Category 2 (≥6-15 ng/L) | n=2 / 252 | 1.26 (0.23– 6.95) |  |  |
| Category 3 (≥ 16 ng/L) | n=4 / 101 | 5.29 (1.66 – 16.87) | **0.014** |  |
|  |  |  |  |  |
| Category 2 & 3 combined | n=6 / 350 | 2.64 (0.93 – 7.47) | 0.067 |  |
|  |  |  |  |  |
| Troponin I ng/L (continuous) | n=14/1935 | 2.64 (1.03-1.60) | **0.026** |  |
|  |  |  |  |  |
| **Mortality by all other causes**  **(excluding cardiac mortality)** |  |  |  |  |
| Category 1 (<6.0 ng/L) ref^¶^ | n=28 / 1562 |  |  |  |
| Category 2 (≥6-15 ng/L) | n=6 / 248 | 0.70 (0.26– 1.84) |  |  |
| Category 3 (≥ 16 ng/L) | n=2 / 103 | 0.75 (0.17 – 3.32) | 0.512 |  |
|  |  |  |  |  |
| Category 2 & 3 combined | n=8 / 351 | 0.71 (0.29 – 1.71) | 0.453 |  |
|  |  |  |  |  |
| Troponin I ng/L (continuous) | n=36/1913 | 0.91 (0.73-1.15) | 0.463 |  |
| **5-10 years** |  |  |  |  |
| **Cardiac mortality ^3^** |  |  |  |  |
| Category 1 (<6.0 ng/L) ref^¶^ | n=7 / 1583 |  |  |  |
| Category 2 (≥6-15 ng/L) | n=2 / 252 | 1 .51 (0.32– 7.12) |  |  |
| Category 3 (≥ 16 ng/L) | n=2 / 103 | 2.51 (0.48 – 13.07) | 0.271 |  |
|  |  |  |  |  |
| Category 2 & 3 combined | n=4 / 355 | 1.88 (0.53-6.56) | 0.321 |  |
|  |  |  |  |  |
| Troponin I ng/L (continuous) | n=11/1938 | 1.11 (0.84-1.46) | 0.445 |  |
|  |  |  |  |  |
| **Mortality by all other causes**  **(excluding cardiac mortality)** |  |  |  |  |
| Category 1 (<6.0 ng/L) ref^¶^ | n=81 / 1509 |  |  |  |
| Category 2 (≥6-15 ng/L) | n=22 / 232 | 1.26 (0.77 – 2.07) |  |  |
| Category 3 (≥ 16 ng/L) | n=4 / 101 | 0.70 (0.25 – 1.93) | 0.969 |  |
|  |  |  |  |  |
| Category 2 & 3 combined | n=26 / 333 | 1.12 (0.70-1.77) | 0.624 |  |
|  |  |  |  |  |
| Troponin I ng/L (continuous) | n=107/1842 | 1.00 (0.89-1.12) | 0.936 |  |
|  |  |  |  |  |
| ^1^Competing risk models adjusted by age, sex, BMI, smoking history, stroke event, myocardial infarction event (previous or incident), hypertension(self-reported), coronary heart disease, diabetes and spirometry categories. ^2^ The following ICD 10 codes were included in that specific variable: I07.1, I11.0, I13.2, I21.0, I21.4, I21.9, I22.9, I25.1, I25.10, I25.5, I25.9, I42.0, I42.9, I46.1, I46.9, I48.9, I49.0, I49.9, I50.19, I50.9, I51.9.  ^¶^ Categories of cTnI, as defined by the following concentrations: <6.0 ng/L, ≥6-15 ng/L, ≥16 ng/L, and continuous values (log_2_ transformed). | | | | |
